# Recent Development of Multifunctional Sensors Based on Low-Dimensional Materials

**DOI:** 10.3390/s21227727

**Published:** 2021-11-20

**Authors:** Qian Xu, Yang Dai, Yiyao Peng, Li Hong, Ning Yang, Zhiqiang Wang

**Affiliations:** Information Science Academy of China Electronics Technology Group Corporation, Beijing 100086, China; xuqian199004@163.com (Q.X.); daiyang_2000@sohu.com (Y.D.); pengyiyao924@163.com (Y.P.); hongli1993@163.com (L.H.); yangning8848@163.com (N.Y.)

**Keywords:** multifunctional sensor, low-dimensional material, smart systems, conductive composite, self-power, piezotronic, piezo-phototronic

## Abstract

With the demand for accurately recognizing human actions and environmental situations, multifunctional sensors are essential elements for smart applications in various emerging technologies, such as smart robots, human-machine interface, and wearable electronics. Low-dimensional materials provide fertile soil for multifunction-integrated devices. This review focuses on the multifunctional sensors for mechanical stimulus and environmental information, such as strain, pressure, light, temperature, and gas, which are fabricated from low-dimensional materials. The material characteristics, device architecture, transmission mechanisms, and sensing functions are comprehensively and systematically introduced. Besides multiple sensing functions, the integrated potential ability of supplying energy and expressing and storing information are also demonstrated. Some new process technologies and emerging research areas are highlighted. It is presented that optimization of device structures, appropriate material selection for synergy effect, and application of piezotronics and piezo-phototronics are effective approaches for constructing and improving the performance of multifunctional sensors. Finally, the current challenges and direction of future development are proposed.

## 1. Introduction

The rapid development of emerging technologies, such as smart portable devices, internet of things, human-machine interface, and wearable electronics, is improving people’s lives revolutionarily [1,2,3,4]. These advanced technologies put forward higher requirements for sensing various kinds of signals [1,5,6,7], such as pressure, touch, strain, light, temperature, and gas. A single type of signal is often insufficient to accurately recognize human actions or environmental situations. More accurate information acquirement relies on monitoring multiple parameters of humans and environments [5,6,8]. For example, a mechanical sensor could be adopted to detect the airflow that blows it, but it cannot distinguish human breathing from a hair dryer or an ordinary gust of wind. If temperature and gas (humidity) sensors are also used synergistically, the action of human breathing will be identified precisely. Furthermore, information expression and energy output through electrical, optical, or thermal manner are of great significance to active behavior control and self-powered systems [4,9,10], which are necessary components to intelligent robots and distributed sensor networks. At the same time, with the ongoing scaling of smart systems, it is very important to compress the volume occupation of each device [11,12,13]. Thus, the versatile functions of sensing and even energy supplying and action are highly desired to be integrated monolithically in a single device [6,14,15].

Due to their nanoscale size, high dispersibility or ductility, and excellent electrical properties, multiple electronic devices based on low-dimensional materials have been demonstrated, such as sensors [16,17,18,19], field-effect transistors (FETs) [11,13,16,20], solar cells [21,22,23], and memory units [24,25,26]. Their diverse and excellent properties and the flexible strategies of device fabrication render them advantageous to build high-performance and multifunctional micro/nano devices. In recent years, unique fabrication methods, novel device designs, and fresh sensing mechanisms have been reported, and low-dimensional materials are the basic and crucial components in these works. Although the technical maturity and overall performance are often inferior to the standard for commercial products, there are many unique advantages that belong to low-dimensional materials only. Firstly, compared to traditional or bulk materials, the primary distinction of low-dimensional materials is the nanoscale sizes. Due to this advantage, low-dimensional materials can be evenly mixed in matrixes, and very little addition of them will modulate the whole performance significantly. Secondly, nanoscale sensors can be assembled with irreplaceable advantages and great potentials on integrated systems and smart applications. Thirdly, the continuous development of nano fabrication technologies make various novel device designs realizable, and these fresh methods and strategies greatly promote the frontier of multifunctional sensors. All above merits attract enormous efforts to explore and develop multifunctional sensors based on low-dimensional materials. In order to summarize the attractive research achievements and inspire future researches in the field, the recent developments are systematically presented in this review.

According to the morphology and dimensions, low-dimensional materials can be divided into three categories, which are 0-dimensional (0-D), 1-dimensional (1-D), and 2-dimensional (2-D) materials. The distinguished features between 0-D, 1-D, and 2-D materials render them suitable for different sensing mechanisms, which are applicable to different device architectures and sensing functions. For example, 0-D materials are usually easy to be dispersed in matrix for fabricating conductive composite [14,27,28], and small additions of them often provides great performance enhancement that is resulted from optimization of the active composite structure or the sensing mechanism [15,29,30]. Thus, they are widely adopted in resistive, capacitive, and self-powered sensors that are sensitive to mechanical and environmental stimulus. On the other hand, a great deal of research interest has recently been attracted by piezotronic and piezo-phototronic effect [6,31,32,33,34], which is based on the coupling between piezoelectric effect and electric/photo-electric properties of semiconductors [35]. Through the attractive coupling, the generation, separation, recombination, diffusion, and transport of carriers are modulated by strain/pressure-induced piezopotential [34,36]. This mechanism not only improves the performance but also endows the devices with integrated mechanical sensing ability, offering a new pathway to multifunctional sensors. However, effective piezopotential from piezoelectric monocrystal or uniformly orientated piezoelectric polycrystal is indispensable, consequently hampering the application of 0-D materials in this type of device. As for 1-D and 2-D materials, attributed to the single-crystal nature [6,18,19,36,37,38,39], the piezoelectric semiconductors among them are capable of generating piezopotential for modulating sensing performance and introducing perception of mechanical signals [10]. Thus, besides the traditional resistive, capacitive, and piezoelectric sensors, the category of 1-D and 2-D materials-based multifunctional sensors is greatly expanded by piezotronic and piezo-phototronic devices.

Generally, in this review, on the basis of classification by 0-D, 1-D, and 2-D, the corresponding sensors are further divided by their main functions and organized according to the specific role of the low-dimensional materials in these sensors in order to clearly summarize and elaborate the device structures and working mechanisms.

In details for 0-D material multifunctional sensors, they are categorized into two groups based on their primary sensing functions. One group mainly includes sensors that detect various mechanical stimulus. This group is subdivided further because 0-D materials mainly serve two purposes in the corresponding sensors: the necessary active components for realize sensing function and the additions to improve performance. The other group mainly consists of sensors able to detect environmental information. Because these environment sensors are almost based on conductive composites or versatile materials, they usually also have ability to perceive mechanical signals. In each section, the corresponding sensors are systematically arranged and presented according to the transmission mechanisms. The 1-D material multifunctional sensors part is organized based on similar framework with 0-D material part and based on the same principle.

In the 2-D material multifunctional sensors part, the corresponding sensors can be directly categorized depending on the devices architectures and working mechanisms. Similar with the 0-D and 1-D materials, 2-D material-based conductive composites are studied widely, and these composite-based devices are macroscopical and basically belong to resistive sensor. On the other hand, single 2-D nanoflake or heterojunction device is an emerging focus area for sensor research [40,41,42]. These micro/nano-scale multifunctional devices usually follow piezotronic and piezo-phototronic effect, where tremendous fascinating achievements have been reported.

## 2. Multifunctional Sensors Based on 0-D Materials

0-D materials usually refer to nanoparticles and quantum dots as well as their cluster [17], including metal, carbon, and semiconductors. These 0-D materials have been widely used as sensitive components, decorating elements, and conductive fillers in various kinds of sensors due to their dispersibility, a high surface-area-to-volume ratio, as well as good electrical characteristics.

When acting as active materials in multifunctional sensor, these 0-D components could change the conductive properties of themselves or modulate the conductive pathways made up of other materials, according to the applied stimulus and sensor structure. For example, a conductive channel made of 0-D semiconductor nanoparticles [43] can respond to light illumination with photon energy larger than the band gap through photocurrent, and for the van der Waals heterostructure (vdWH) photodetectors consisting of 0-D quantum dots and 2-D channel [44,45], photo-induced carriers transfer from the quantum dots to the channel could cause performance improvement by several times or even magnitudes compared with the counterparts without quantum dots. Besides, conductive 0-D materials can also involve structural change of current paths in conductive composite; for example, reconstruction of the current paths could be induced by contract or separation between adjacent conductive nanoparticles attached to the 3D construction [14,16,17,27] or flexible fiber [17], caused by mechanical stimulus.

### 2.1. Multifunctional Mechanical Sensors

Mechanical signals, such as tough, press, strain, and shear force, exist extensively in everyday situations. It is a very important function of the skin to sense mechanical signals, and monitoring of mechanical signals are also often indispensable in industry. Thus, mechanical sensors have attracted much attention and become the research focus in the field of human-computer interface, smart electronic skin, health monitoring, and so on. 0-D materials can easily attach or coat on backing materials [17,29,37,46,47,48], such as natural or man-made fibers, planar flexible substrates, 3D porous frameworks, and so on, by sample methods of soaking, spraying, spin-coating, dropping, and others facile processes. This advantage endows 0-D materials great potential for mechanical sensing. In the Section 2.1, we focus on the 0-D material based multifunctional sensors, which are capable of transducing mechanical deformation into electrical signals, and illustrate the role of the 0-D materials in these sensors.

#### 2.1.1. 0-D Materials as Sensing Components

Being embedded in or attached on a deformable matrix, 0-D sensing materials could be employed to cost-efficiently and facilely build a multifunctional sensor. The primary sensing mechanism is the piezoresistive effect, while another recently proposed novel mechanism of piezo-thermic transduction [14] is also illustrated in this section.

**Figure 1:**
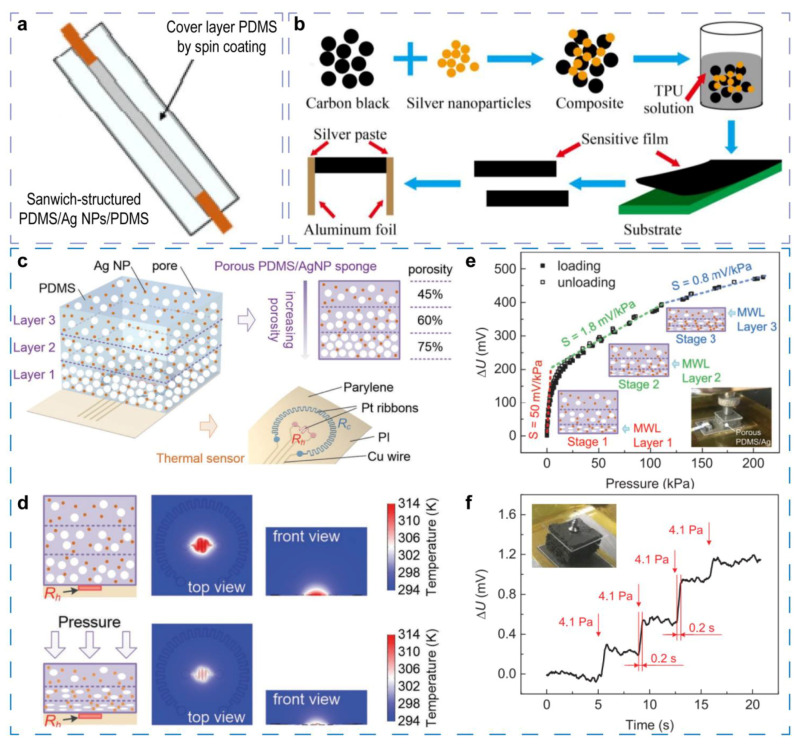
Multifunctional mechanical sensors employing 0-D materials as sensing components. (**a**) A sample and typical sandwich structure example for mechanical sensors. Reproduced with permission. [37] Copyright 2020, Springer Nature. (**b**) Fabrication processes of the sensor based on CB/Ag NPs composites. Reproduced with permission. [48] Copyright 2018, MDPI. (**c**) Schematic diagram of the piezo-thermic transduction mechanical sensor. (**d**) Steady-state temperature field in the sensor simulated by finite element analysis. (**e**,**f**) Pressure sensing performances and the low detection limit of the sensor, respectively; the inset in (**e**,**f**) are the corresponding measurement setups. Reproduced with permission. [14] Copyright 2019, Wiley-VCH.

Piezoresistive composites usually consist of conductive materials and a deformable matrix that could be various flexible materials. When the matrix deform under external stimulus, 0-D conductive materials in the piezoresistive composite are resultantly separated, and the resistance is increased. A sample and typical example [37] is a strain sensor fabricated by drop-casting Ag nanoparticles on polydimethylsiloxane (PDMS) substrate, and the sensor is encapsulated by another PDMS layer to form a sandwich-like structure (Figure 1a). In other reports, Ag nanoparticles could be substitute by other 0-D conductive components, such as carbon black (CB), and other substrate materials as well as device structures, such as PU fiber, 3D PDMS matrix, silk, and so on [17], could be used for better stretchable and sensitivity. This category of sensor can usually capture multiple kinds of mechanical deformation.

A strain and bending sensor is constructed based on CB/Ag NPs composite dispersed in thermoplastic polyurethane (TPU) matrix [48]. To fabricate the sensor, CB is modified by poy(vinylpyrrolidone) (PVP) to attach Ag NPs, then the CB/Ag NPs composite is mixed with TPU solution, and finally the liquid mixture is coated as a film and heated to evaporate solvent (Figure 1b). Compared to the bare CB-based sensor with a gauge factor of 1.12 at 100% strain, the CB/Ag NPs composite-based sensor exhibits a gauge factor as high as 21.12 at the same strain. The integration of Ag NPs generates three junctions of Ag NPs-Ag NPs, Ag NPs-CB, and CB-CB instead of only CB-CB in the bare CB-based sensor. It is proposed by the authors that the different junctions provide more variable resistance, and thus a higher sensitivity is obtained.

Besides higher sensitivity, broader measure range and low detection limit have both been strongly desired. To obtain these desired properties simultaneously, a novel mechanism of piezo-thermic transduction is suggested, which totally distinguishes from the piezoresistance effect [14].

In the sensor based on this novel mechanism, Ag NPs are used as sensing fills to improve heat conductance instead of conductivity [14]. In details, as shown in Figure 1c, the sensor consists of a thermal sensor subassembly and a sponge that is composed of porous PDMS and Ag NPs. The thermal subassembly comprises a polyimide (PI) substrate, two Pt ribbons, four Cu wires, and a parylene film. The two Pt ribbons are derived by a constant temperature difference (CTD) conditioning circuit. When the circuit operates, the center ribbon R_h_ (100 Ω) reaches a higher temperature because of Joule heating, and a heat transfer from R_h_ to the porous sponge is formed (Figure 1d). Because the effective thermal conductivity of the sponge can be improved by applied pressure, the temperature of R_h_ will be decreased, and the output voltage U will be increased by the CTD feedback circuit. For sensing pressure in a different range, the PDMS sponge possesses three layers with the porosity of 45%, 60%, and 75%, respectively. The graded-porosity PDMS is fabricated by a sacrificial template method in which citric acid monohydrate (CAM) is used as the template, and the three varied porosities are obtained by the controlling volume ratio of PDMS and CAM. The gradient-increasing porosities render the composite sponge’s gradual decline of Young’s modulus; thus, each of the three layers mainly responds to a different pressure range, and three distinct sensing stages are observed as shown in Figure 1e. It is worth noting that the thermal conductivity of bare PDMS is only about 0.177 W m^−1^ K^−1^ and is too low to obtain high sensitivity. To enhance the thermal conductance, Ag NPs are embedded into PDMS sponge as filler due to the excellent dispersibility and isotropic thermal conductivity. Furthermore, appropriate addition of Ag NPs also leads to a lower Young’s modulus. According to the theoretical analysis, the higher thermal conductivity and lower Young’s modulus improve the sensitivity synergistically, but the measurement range decreases when the Young’s modulus decreases. Thus, the Ag NPs volume ratio of 2 vol% is selected, and the thermal conductivity is enhanced to 0.243 W m^−1^ K^−1^. Attributed to the ingenious device designing and optimizing, the sensing range is up to 200 kPa, and the highest sensitivity is about 50 mV kPa^−1^, obtained in a pressure range of less than 4 kPa (Figure 1e). To obtain the detection limit of this sensor, four small weights are successively put onto the sensor that is covered by a thin slide (the inset in Figure 1f), and the applied pressure is increased by the step of 4.1 Pa. As shown in Figure 1f, a detection limit and resolution as low as 4.1 Pa can be achieved. In addition, the annular ribbon (Rc with a resistance of 1000 Ω) keeps a temperature approximately equal with the ambient, allowing simultaneous sensing of pressure and temperature conducted by the CTD circuit. Owing to the low detection limit and the wide measuring range, human actions from a subtle wrist pulse to foot-stepping pressure can be monitored. This work proposes a new sensing principle for mechanical stimulus that is different from usual piezoresistive effect, and a new function of 0-D material filler in multifunctional sensors also is revealed.

#### 2.1.2. 0-D Components for Performance Enhancement

The nanoscale size of 0-D materials offers efficient dispersibility on the surfaces of other materials, and remarkable performance enhancement could be obtained by a very small addition of 0-D materials, owing to the resultant significant changes in the device structure and working mechanism. In the Section 2.1.2, we will not only illustrate 0-D material enhanced traditional piezoresistive sensors, which were involved in Section 2.1.1, and capacitive sensors [30] but also recently emerging self-powered sensors conducted by piezoelectric and triboelectric effects [49,50].

Recently, researchers’ attention has been attracted by multifunctional sensors capable of detecting tiny mechanical signals by which various kinds of human activities can be revealed. It has been reported that appropriate addition of 0-D materials will improve the sensitivity to subtle mechanical signals and promote the application of multifunctional sensors.

**Figure 2:**
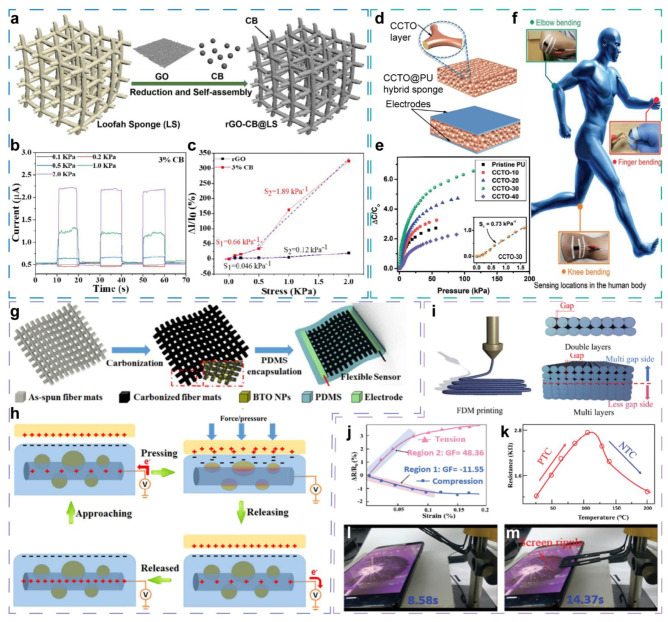
(**a**–**h**) Multifunctional sensors enhanced by 0-D components, and (**i**–**m**) the integrated sensor-actuator with sensibility to mechanical stimulus and temperature. (**a**) Fabrication processes and device structure of the rGO@LS conductive composite resistive sensor enhanced by CB nanoparticles. (**b**) The current response of rGO-CB@LS sensor under different pressure. (**c**) The normalized current response of the sensors with CB addition of 0% and 3%, respectively. Reproduced with permission. [29] Copyright 2020, American Chemical Society. (**d**) Schematic illustration of structure of CCTO@PU hybrid sponge and the capacitive sensor. (**e**) The normalized capacitance response curves with different CCTO loading percentages. The inset exhibits the sensitivity of CCTO-30-based sensor below 1.6 kPa. (**f**) Schematic illustration of multifunctional application for detecting human actions. Reproduced with permission. [30] Copyright 2020, Wiley-VCH. (**g**) Fabrication processes and device structure of the PAN-C/BTO composite sensor. (**h**) Self-powered working mechanism of PAN-C/BTO composite sensor. Reproduced with permission. [50] Copyright 2015, American Chemical Society. (**i**) Assembly processes and the gradient gap structures of the integrated sensor-actuator. (**j**,**k**) Strain and temperature detection of the sensor-actuator, respectively. (**l**,**m**) The process of mimicking the finger action of touching screen. Reproduced with permission. [27] Copyright 2020, Wiley-VCH.

Minghui Cao et al. fabricated an ultralight 3D hybrid piezoresistive sensor with loofah sponge (LS) as the deformable framework [29]. The conductive component is reduced graphene oxide (rGO) that is prepared by reduction of graphene oxide (GO) precursor and a subsequent self-assembling process, and carbon black (CB) was introduced to modify the inner conductive path and optimize the sensitivity. The schematic diagram of the preparation process is shown in Figure 2a. With the addition of CB, the conductivity was improved, and the charge transport paths in 3D LS were changed significantly compared to the counterpart without CB. It is worth noting that the current decreases firstly under tiny stress and then increases with larger applied pressure instead of a monotonically increasing current as expected, as shown in Figure 2b. This abnormal current is different from the general piezoresistive sensors. It is proposed that CB nanoparticles form scattered islands with an appropriate amount of CB; restricted by the insufficient content of CB, CB islands are isolated from each other, and thus the new paths formed by CB are not continuous on LS. Additionally, the current of this sensor can be defined as I = Ic + Ir, where Ic and Ir are the current provided by CB islands and the contact of rGO, respectively. When a tiny stress is applied, the partial destruction of CB islands emerges and Ic decreases, while Ir increases very slightly; thus, the total current I exhibits a significant decrease. This phenomenon improve the sensitivity of the sensor to small stress. As the applied stress increases continuously, contact between rGO and CB will lead to an increase in current. Thus, the abnormal reduction in current is eliminated under a specific pressure defined as “turning stress”, according to CB content. Benefited from the advantages by introducing CB, thr rGO-CB-based sensor with 3% CB showed a greater current, and the sensitivity was improved to 0.66 KPa^−1^ and 1.89 KPa^−1^ in the pressure range of 0–0.5 KPa and 0.5–2.0 KPa (Figure 2c), respectively, with a “tuning stress” of 0.5 KPa. While the rGO-based sensor exhibits the current of only about 120 nA, and small stress ranging from 0 to 0.5 KPa can hardly be observed due to the negligible current change. In addition, the response time of the hybrid sensor was reduced from 0.73 to 0.42 s after the addition of 3% CB, while the recovery time was also shortened from 0.48 to 0.29 s. This multifunctional rGO-CB@LS sensor was applied to sense exhaling, blowing, speaking, and finger pressuring, and these results demonstrate the ability of rGO-CB@LS sensor to sense subtle actives with pivotal inclusion of 0-D CB.

Capacitive sensor is another primary device type to detect mechanical signals with high sensitivity, excellent linearity, and low hysteresis. Commonly, in the structures of capacitive sensors, low-dimensional materials tend to be used in flexible or transparent electrodes owing to their conductivity and dispersibility, and insulating low-dimensional materials are often overlooked especially in flexible capacitive sensors. Recently, through composition and structure optimization of the dielectric [30], a capacitive multifunctional sensor with enhanced sensitivity was developed by Ashok Chhetry et al. This sensor is fabricated by sandwiching the optimized dielectric between two same electrodes (Figure 2d). To fabricate the dielectric, calcium copper titanate (CCTO) nanoparticles are surface-modified by 3-aminopropyl triethoxysilane (APTES) and robustly fixed onto the surface of a porous PU sponge by a dip-coating process. The CCTO@PU hybrid sponge dielectric provides a dielectric permittivity as high as 167.05 and a low loss tangent of 0.71. Additionally, the sponge structure provides an ultralow compression modulus (27.83 kPa). The remarkable dielectric and electromechanical properties render the capacitive sensor’s sensitivity as high as 0.73 kPa^−1^ in the pressure range less than 1.6 kPa (Figure 2e). When attached to a volunteer’s fingers, joints, and larynx, as shown in Figure 2f, the sensors form a system that can recognize grasping behavior and the different weights of grasped objects, detect joint bending to reflect human actions, and monitor small activities, such as phonation and swallowing.

Some other researches adopt 0-D materials as additions to introduce piezoelectric effect into multifunctional sensors. Self-powered mechanical sensor based on piezoelectric and triboelectric effect is a new pathway for integration of sensing and energy supply [49,50]. Recently, 0-D piezoelectric nanoparticles are employed to fabricate the sensors conducted by the self-power principle.

The piezoelectricity of lead halide perovskite materials has been verified [51,52], and attentions have been drawn for their rapid development in constructing high-performance devices. Geng Chen et al. fabricated a hybrid piezoelectric nanogenerator that is composed of FAPbBr_3_ nanoparticles and PDMS matrix [49]. Owing to the piezoelectric charge coefficient (d_33_) as high as 25 pmV^−1^, the outstanding output performances of 8.5 V voltage and 3.8 μA cm^−2^ current density are achieved, and the output signals reflect the applied stress.

Gengrui Zhao et al. doped barium titanate nanoparticles (BTO NPs) into the carbonized electrospun polyacrylonitrile (PAN-C) nanofiber film in order to introduce piezoelectric effect and improve the self-powered sensing [50]. As shown in Figure 2g, the PAN-C/BTO-based sensors are fabricated via a facile electrospinning, carbonization, and encapsulation process, and the as-fabricated sensors integrate piezoresistive, triboelectric, as well as piezoelectric effect synergistically. The sensors have two main working modes that are flexible-bending sensing and self-powered pressure sensing. Base on the impedance increase of the conducting nanofiber film under stretching deformation on account of bending, flexible-bending sensing is realized, with a gauge factor of 1.12 deg^−1^ from 58.9° to 120.2° and a working range of 28~150°. However, for flexible-bending sensing, this is a very common sensing principle in the stretching-releasing process for two-terminal resistance device. Notably, the PAN-C/BTO sensors can also work for self-powered pressure sensing as a single-electrode triboelectric nanogenerator (SE-TENG), and the integration of BTO nanoparticles is important for the synergy of piezoelectric and triboelectric effect that results in a higher sensitivity. In this sensing mode, the flexible encapsulating PDMS works as a dielectric layer for producing electrical charges by the contact electrification, and the carbonized PAN-C nanofiber layer acts as an electrode for collecting those charges and conducting current. The working mechanism of self-powered pressure sensing is depicted in Figure 2h. When an active object is pressed on the PDMS, triboelectrification occurs at the interface. With an increased pressure, the contact area would also increase due to the microcosmic deformation of PDMS, leading to more charge separation at the contact interface until the pressure reaches a certain extent, and the contact area cannot be increased anymore. When the active object departs from PDMS, equivalent positive charges will be induced through the conductive PAN-C nanofiber layer due to the electrostatic induction effect. Interestingly, the authors compare output signals of PAN-C- and PAN-C/BTO-based sensors, and the remarkable signal enhancement is observed by embedding BTO nanoparticles in the PAN-C nanofiber. Owing to the inclusion of BTO, a high sensitivity of 1.44 V·N^−1^ is observed in the range of 0.15~25 N, which is 2.4 times higher than that of the undecorated PAN-C sensor. The enhancement of output performance is owed to the piezoelectric effect of BTO nanoparticles. The direction of the piezoelectric polarization of BTO nanoparticles in this system is perpendicular to the axis of PAN-C. It is analyzed and verified by finite-element simulation that the negative charges on the sensor surface and the positive charges in PAN-C increase accordingly with the increased press, attributing to coupling of triboelectric charges and piezoelectric charges. Based on the outstanding flexibility and versatility, the PAN-C/BTO-based sensor could detect bending and pressure simultaneously. For example, when attached on knuckles, the sensors could capture subtle motions of fingers; and when stuck on finger pulps, the sensors could detect the touch movement and the strength of press. A sensing system could be built conveniently by distributing the PAN-C/BTO sensors on human body, such as a gesture-sensing system comprised of 10 sensors attached to each finger. With the combination of bending and tough sensing, gesture language, such as “good”, is recognized by the sensing system. In addition, human health-related motion, such as swallowing and gait as well as material shape and geometry, could be monitored by the sensing system based on PAN-C/BTO sensors. Through the modification of 0-D BTO, multiple sensing mechanisms are coordinated in one sensor, facilitating multifunctional sensing and more accurate motion recognition.

Piezoelectric potential generated by a nanoparticle is weak, and the subtle potential of each nanoparticle needs to be superposed by aligning the dipole orientation. As a result, the features of nanoscale size and random dispersion of 0-D materials actually limited the development of piezoelectric sensors. Although there have been many reports that illustrate the outstanding performance and versatility of devices based on piezoelectric thin films and bulk materials, more efforts should still be made to develop facile synthesis and accurate assemble method to improve piezoelectric potential output for nanoparticles-based mechanical sensors.

### 2.2. Environmental Sensors with Monolithically Integrated Strain/Pressure Detection

In this section, recent multifunctional sensors capable of detecting mechanical stimulus and environmental variation are presented and classified according the sensing mechanism by a sequence of structure-dependent piezoresistance [27], thermal resistance [53], gas molecular modification [53], as well as integration of photovoltaic, piezoelectric, and pyroelectric effects [15].

As illustrated the Section 2.1, contact and separation of conductive fillers modulate the resistance of sensors. The same mechanism is also employed by temperature sensing [8], and thus, mechanism signals and ambient temperature can be monitored by one sensor. For example, it has been reported that a metal-organic frameworks-derived porous carbon and PDMS composite-based sensor is capable of detecting pressure, strain, and temperature due to the contact area change between porous carbon particles that involves PDMS deformation originating from external force and thermal expansion [8]. The porous carbon and PDMS composite sensor exhibit a high sensitivity of 15.63 kPa^−1^ and a fast response time of 65 ms for pressure as well as 0.11 °C^−1^ sensitivity and about 100 ms response time for sensing temperature.

Very recently, combining structure-dependent resistance and temperature-dependent resistance, an integrated sensor–actuator, which is capable of sensing mechanical stimulus and temperature, was fabricated form composites of 0-D CB and polylactic acid (PLA) by 4D-printing method [27]. A unique, bioinspired, and gradient gap structure from bottom to top is obtained by the 4D-printing method, as shown in Figure 2i. As a mechanical sensor, the gradient gaps are smaller under a force from the top to the bottom (compression) and larger under a reversed force (tension), and a gauge 0–0. factor of 48.36 in 7% strain is obtained (Figure 2j). As a temperature sensor, the resistance increases with raised temperature from 20–120 °C and decreases from 120–200 °C (Figure 2k), and the sensitivity of 1.90 × 10^4^ and −2.25 × 10^4^ ppm °C^−1^ is obtained, respectively. Besides the structure deformation, the resistance change by mechanical and temperature stimulus can be mainly attributed to the electron tunneling effect between CB nanoparticles. In addition, the active touch to the object is also demonstrated (Figure 2l,m), and the touch force feedback can be captured by the resistance change.

Humidity sensing is also expected in applications of environmental monitoring and control as well as health care. Hanbin Liu et al. construct a multifunctional sensor [53], which possesses the ability of detecting strain, environmental pressure, temperature, and humidity though spraying the composite of CB and rGO onto paper. Benefited from the hybrid low-dimensional active components and the hierarchical structure comprising porous and micro-gaps, the sensor is multifunctional, and good performances were achieved. For strain sensing, the GF is 14.6 and 1.8 for compression and tension, respectively, with a response time about 340 ms. The sensor also exhibits the function to reflect variation of environmental pressure and temperature. As for detecting environmental pressure, there are two liner areas in the measured pressure region. The linear slope is 0.59 and 0.09 kPa^−1^ in the range of 0–50 kPa and 50–250 kPa, respectively. When working as a temperature sensor in the measured range of 20–60 °C, the sensor displays a negative temperature coefficient (NTC)-resistance characteristic with the linear fitting slope of 0.6 °C^−1^. When placed under different humidity conditions, the sensor resistance approximately shows a linear increase with increased humidity, and a slope of 2.04 is observed.

Photovoltaic, piezoelectric, and pyroelectric effect have been combined together to construct a multifunctional sensor based on BTO nanoparticles [15]. For constructing the sensor, BTO nanoparticles are sintered and sanded to form BTO ceramic wafers with ~280 μm thickness, and after polarization, ITO and Ag electrodes are deposited on the top and the bottom, respectively. Owing to photovoltaic, piezoelectric, and pyroelectric effects that co-exist in BTO and the regulated carrier separation and transport by piezoelectric and pyroelectric potential, a larger electrical output is obtained, and photodetection, pressure detection, and temperature sensing are integrated monolithically. The enhanced sensitivity of photodetection, pressure detection, and temperature sensing are 0.42 nA/(mW/cm^2^), 1.43 nA/kPa, and −8.85 nA/K, respectively.

## 3. Multifunctional Sensors Based on 1-D Materials

1-D materials possess high length-diameter ratio, including nanorods, nanowires, nanotubes, and so on. These materials could provide outstanding flexibility, transparency, conductivity, and piezoelectricity. Owing to these remarkable properties, resistive, capacitive, and emerging self-powered sensors based on 1-D materials are widely researched [54,55,56,57,58,59,60]. Furthermore, as stated previously, owing to the ability of generating effective piezopotential under strain or pressure, 1-D piezoelectric nanowires with monocrystal nature are employed to construct piezotronic and piezo-phototronic devices, which integrate environment detection with mechanical signals sensing [34,35,36,39,61].

### 3.1. Multifunctional Mechanical Sensors

1-D materials can be used widely to construct various parts in multifunctional mechanical sensors, such as all-fiber substrates, composite conductive fibers, as well as high robust flexible electrodes. Different from 0-D materials, 1-D materials are flexible and could be directly integrated in clothing. In addition, 1-D materials-based devices could provide excellent pliability and ductility. Similar to 0-D materials, 1-D materials can also be either attached on or integrated with other materials as active or decorating components. When coated on or embedded in matrix material, 1-D materials could form not only isolated particles or thin film but also fiber networks that are generally more flexible and stretchable.

#### 3.1.1. 1-D Conductive Materials as Sensing Components

**Figure 3:**
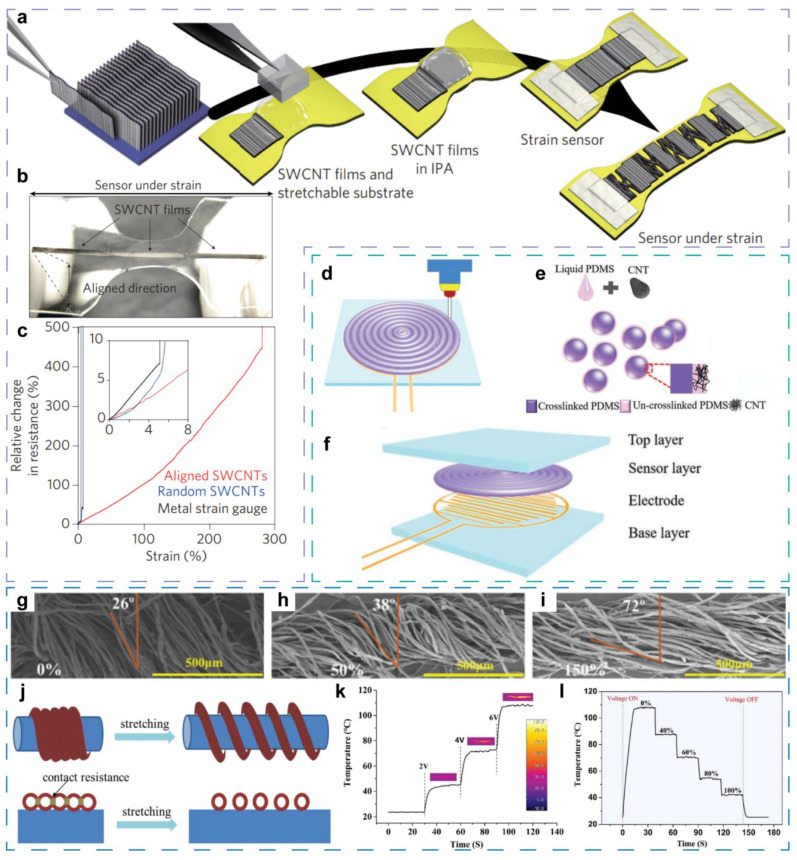
Multifunctional mechanical sensors employing 1-D conductive materials as sensing components. (**a**) Fabrication steps of the SWCNT strain and bending sensor. (**b**) Photograph of the SWCNT sensor. (**c**) Relative change in resistance of aligned under strain up to 280%. The inset is magnification of the low-strain region, showing the details for sensor based on random SWCNTs and conventional metal thin film. Reproduced with permission. [62] Copyright 2011, Springer Nature. (**d**) Schematic of 3D-printing method for the piezoresistive sensor based on PDMS MPs and CNTs that mimics the texture and sensitivity of human skin. (**e**) The new type of 3D-printing ink for fabricating the sensor, containing PDMS MPs, uncured PDMS precursor, and CNTs. (**f**) Structure diagram of the PDMS/CNT composite sensor. Reproduced with permission. [58] Copyright 2019, Wiley-VCH. (**g**–**i**) SEM image of the Ag NWs/CPY sensor under strain of 0%, 50%, and 150%, respectively. (**j**) AgNWs/CPY model for illustrating the sensing mechanism. (**k**) Temperature of the AgNW/CPY composite under increased voltage and without strain. (**l**) Temperature of the AgNW/CPY composite under voltage of 6 V and with increased strain. Reproduced with permission. [65] Copyright 2019, Royal Society of Chemistry.

Similar to 0-D materials, a typical 1-D material piezoresistive sensor is fabricated by integrating 1-D conductors with a flexible matrix. Early in 2011 [62], Yamada T et al. proposed a stretchable sensor made up of well-aligned, single-walled carbon nanotubes (SWCNTs) and a PDMS film with 1-mm thickness (Figure 3a,b). The alignment orientation of SWCNTs is perpendicular to the strain axis. This orientation is beneficial for improving the stretchability, and as a result, the strain limit of SWCNTs sensors is up to 280% (Figure 3c). The stretchable sensor, which is capable of sensing strain and bending, could be integrated into clothing or attached to the body for detecting human motions, such as breathing, phonation, gesturing, and so on.

Additionally based on aligned CNTs, Seongwoo Ryu et al. developed an ultra-high stretchable sensor that employs Ecoflex as flexible substrate [63]. In this work, the orientation of aligned CNTs is parallel with the strain axis, and Ecoflex is pre-strained to improve the stretchability. Attributed to the unique strategy, the ultra-high strain limit of 960% and the outstanding sensitivity of 64 are achieved simultaneously. For optimizing and balancing sensitivity, working range, device function, and preparation cost, continuous efforts have been made to explore structure designs and preparation methods.

Combining 3D-printed ductile framework and CNTs-based conductive network, a novel piezoresistive sensor that mimics the texture and sensitivity of human skin was designed [58]. The 3D framework consists of interconnected PDMS microspheres (MPs), and CNTs cover on the surface of PDMS MPs. The unique device structure is depicted in Figure 3f, which is fabricated by a new type of 3D-printing ink containing PDMS MPs, uncured PDMS precursor, and CNTs, as well as a water-based medium (Figure 3d,e). Owing to the ability of printing customized shapes by 3D-printing technology, the texture of PDMS MPs-CNTs active layer is designed to be similar to the human fingerprint. After printing the active layer on the interdigital electrode, the sensor is encapsulated by the PDMS membrane. Besides simulating the texture of a fingerprint, the sensitivity is very important for electronic skin. Upon applied press, the resistance between the active layer and the electrode is modulated, and current path in active layer is also reconstructed due to deformation of the 3D PDMS MPs-based skeleton. With the increased pressure, the interspace between the CNTs and the interspace between the CNTs and the interdigital electrodes are compressed, resulting in increased contact area and decreased resistance. An ultrahigh sensitivity of 2.08 kPa^−1^ is obtained at a tiny pressure of 0.12 kPa, showing the potential to detect light touch as sensitively as human skin. The feeling to sliding force is another character of human skin, which also is mimicked by the PDMS MPs-CNTs-based sensor. When a nylon probe slides on the surface with a normal force of 0.5 N, the resistance becomes reduced because the sliding force causes contact between CNTs on the PDMS MPs surfaces. In addition, attributed to the increasement of contact areadependent on the deformation of the PDMS MPs-based matrix, PDMS MPs-CNTs sensor is capable of detecting joint bending. Furthermore, the instant response curve of PDMS MPs-CNTs sensor exhibits a fast respond time of 50 ms, which is comparable to the Meissner corpuscle conduction velocity (about 35–70 ms) of human skin. Duo to the outstanding sensitivity, response time, and multifunction and mechanical properties that mimic human skin, this work reveals the great potential of 1-D CNTs and other similar materials for multifunctional mechanical sensing.

Songfang Zhao et al. reported a piezoresistive sensor comprising CNTs, Ag NPs, and hydroxyl-poly-(styrene-block-butadiene-block-styrene) (OH-SBS) matrix [64]. Both of CNTs and Ag NPs are embedded into the flexible matrix as conductive filler. Owing to the conductive bridges of 1-D CNTs that construct effective current pathways among Ag NPs, the composite possesses a high conductivity of 1228 S cm^−1^, and the sensor exhibits an excellent gauge factor of 26,500, with the strain limit of 540%.

Ag nanowires are another typical 1-D material that are widely adopted for detecting mechanical signals because of the great ductility, conductivity, and process compatibility, which are highly desirable for constructing mechanical sensors. At the same time, more beneficial features, such as heating, are highly expected for wearable health applications.

Min Zhao et al. fabricated a stretchable multifunctional sensor combining strain sensing and heating capabilities [65], which are based on cotton/polyurethane core-spun yarn (CPY) and Ag nanowires. The CPY consists of polyurethane (PU) monofilament fiber and cotton yarn. The PU monofilament is inner core fiber, and cotton yarn helically wraps around the PU fiber (Figure 3g). After synthesizing the Ag nanowires by a facile polyol method, the Ag nanowires are coated on CPY through a sample dip-coating process to assemble a conductive network layer based on Ag nanowires. To improve adhesion of Ag nanowires, the CPY is modified with polydopamine via in-situ polymerization before the dip-coating process. Due to the elastic fiber-based structure, the sensor could be stretched up to 200%. Under increased tensile strain, the winding angle of cotton is increased, as shown in Figure 3h–j, resulting in separation of neighboring cotton fibers, and the contact resistance between Ag nanowires also becomes larger accordingly. The resistance incensement is measured under tensile strain ranging from 0–200%; the resistance shows a faster increase rate with larger strain section because of more separation of adjacent Ag nanowires. As a result, a gauge factor of 4.2 is achieved at the 200% tensile strain, while the gauge factor within 50% strain is 1.6. Although the measurement result exhibits a broad sensing range and a competitive gauge factor, resistance hysteresis is observed during stretching–releasing cycles due to the interaction between the Ag nanowires and the PU material. With 50%, 100%, and 150% strain repeatedly applied, the sensor shows stable and reproducible resistance response during the cyclic tensile strain, revealing the potential for practical applications. The sensor is attached on joints, such as the knuckle, elbow, wrist, and knee of the volunteer, so that the wearable application of motion detecting, health monitoring, human–machine interactions, etc., are demonstrated. Last but not least, as a versatile sensor, the CPY-Ag nanowires sensor also is a wearable heater. When a constant bias voltage (2–6 V in this research) is applied, the Ag nanowires network generated heat at once, and a temperature plateau appears after about 11 s (Figure 3k). When the external voltage turned off, the temperature begins to decline at once. Moreover, applied strain will lead to a decrease of the temperature plateau because of the resistance increase caused by the strain, showing the tenability of the heating performance (Figure 3l). This kind of functional characteristic, which does not belong to the category of sensing but actively influences the external environment, expands the boundary of multifunctional sensors. Other researches also report similar versatility that integrates a heating function into a sensor, utilizing joule heat produced by current though conductors under an external voltage. Although the heating function does not involve a new mechanism, we think that the combination of sensing, which receives external signals, and heating, which could express information to the outside, may be a kind of important attempt toward sense-information-integrated devices.

#### 3.1.2. 1-D Flexible Electrodes for Multifunctional Sensors

High stretchability and flexibility are desired for wearable devices. Compared to patterned electrodes fabricated from nanoparticles and metal wires, 1-D material electrodes usually exhibit better stretchability, flexibility, and transparency with a lower cost.

**Figure 4:**
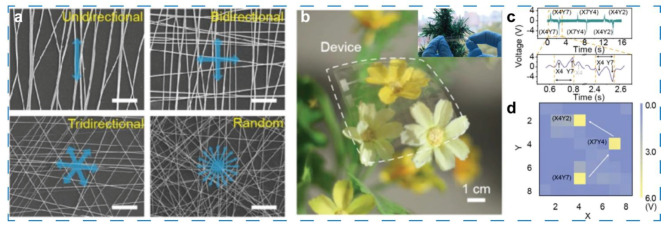
Highly stretchable and transparent Ag-nanofiber electrodes and self-powered sensor based on the electrodes. (**a**) SEM images of PVA nanofibers with different orientation. (**b**) Photograph of the 8 × 8 matrix that is made up of the self-powered triboelectric tactile sensor. The inset is a photograph of the electrodes. (**c**,**d**) Sensor signals when the finger tough the pixel sequentially in the sensor and the corresponding mapping image, respectively. Reproduced with permission. [54] Copyright 2018, Wiley-VCH.

Wang et al. fabricated highly stretchable and transparent Ag-nanofiber electrodes for a self-powered tactile sensor [54]. As the key part, the Ag-nanofiber electrodes exhibit excellent stretchability and transparency, as shown in the inset of Figure 4d. They are prepared by depositing Ag metal on electrospun poly(vinyl alcohol) (PVA) nanofibers and subsequent patterning by photolithography and wet-etching process. The fiber density is optimized to improve the transparency and conductivity; as a result, a low sheet resistance of 1.68–11.1 Ω^−1^ and high transmittance better than 70% are obtained with a fiber density less than 0.5 um^−1^. The influence of the angle between the tension direction and the spinning orientation to stretchability is explored (Figure 4a), and only 10% resistance increase is achieved at 100% strain with a multi-orientation spinning strategy. When applied in self-powered triboelectric tactile sensor as electrodes, the multidirectional Ag nanofiber electrodes exhibit sable conductivity, while the output voltage of the unidirectional Ag nanofiber electrodes-based sensor decreases with the applied strain. In addition, an 8 × 8 matrix (Figure 4b) made up of the triboelectric tactile sensors is constructed to demonstrate the ability of tactile mapping (Figure 4c,d).

### 3.2. Environmental Sensors with Monolithically Integrated Strain/Pressure Detection

**Figure 5:**
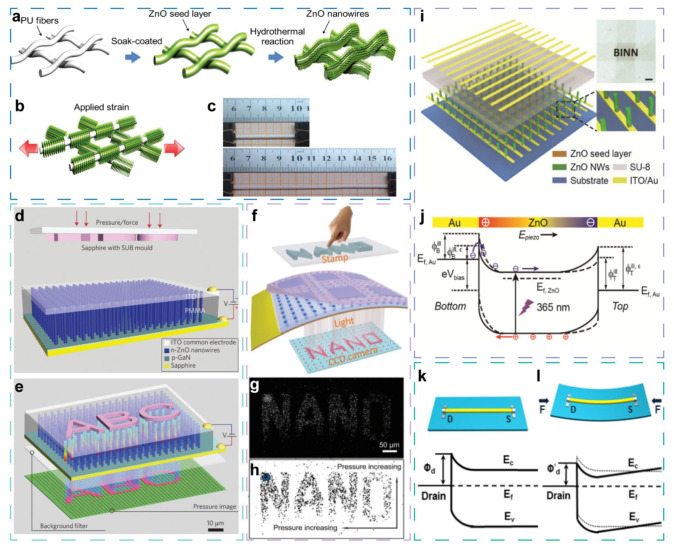
1-D material-based environmental sensors with monolithically integrated strain/pressure detection. (**a**) Key fabrication steps for ZnO/PU fiber based sensor. (**b**,**c**) Schematic diagram and photograph of the ZnO/PU fiber-based sensor under tensile strain, respectively. Reproduced with permission. [56] Copyright 2015, Wiley-VCH. (**d**,**e**) Schematic diagram of device structure and experimental approaches of the LED array based on ZnO NWs for mapping pressure distribution, before (**d**) and after (**e**) applying a pressure by an “ABC” mold. Reproduced with permission. [34] Copyright 2013, Springer Nature. (**f**) Schematic illustration of the sensor based on CdS nanorods. (**g**,**h**) The light-emitting image and the two-dimensional contour map of the LED array under 100 MPa pressure from the “NANO” stamp, respectively. Reproduced with permission. [39] Copyright 2016, Royal Society of Chemistry. (**i**) Schematic structures illustration of the UV based on vertically aligned ZnO nanowires. Inset is an optical image of the device. The scale bar is 1 mm. (**j**) Energy band of Au/ZnO Schottky contact with compressive strains, illustrating working mechanism of the photodetector array. Reproduced with permission. [35] Copyright 2015, Wiley-VCH. (**k**,**l**) Energy band of the Schottky contact of ZnO gas sensor with and without compressive strains in vacuum, depicting the modulation on barrier height of piezotronic effect. Reproduced with permission. [36] Copyright 2015, Elsevier.

Similar to the example of the CB-rGO sensor in the Section 2.2, through combining structure-induced resistive change, pyroelectric effect, and photoelectric effect, Xinqin Liao et al. reported a stretchable sensor with sensing abilities of strain, temperature, and ultraviolet light [56]. The sensor is fabricated by growing ZnO nanowires arrays on the highly stretchable PU fibers through a hydrothermal process, as shown in Figure 5a. The as-grown ZnO nanowires form a thin film covering the PU fiber, and the thin film cracks under applied strain (Figure 5b). Consequently, the resistance decreases with more cracks generated. The GF is 15.2 and 4.1 under the strain below 10% and 10–150%, respectively. The photograph of the ZnO/PU fiber-based sensor under tensile strain is depicted in Figure 5c. When temperature increases, the thermal expansion leads to spontaneous polarization of ZnO NWs, which creates a polarization electric field and generates separated charges in the ZnO NWs, finally resulting in the current increase. In the measured rage from room temperature to 50 °C, the sensitivity of 39.3% °C^−1^ is obtained without applied strain. As a stretchable UV sensor, a high ON/OFF ratio of 158.2 is achieved without applied strain, which is mainly attributed to the surface depletion layer generated by absorbed oxygen molecules. Owing to the multifunction and stretchability, the sensor demonstrated the potential for wearable human actions and environment monitoring.

Distinguishing from the 0-D counterparts, in the family of 1-D multifunctional sensors, there are emerging and important members of the sensors based on piezotronic and piezo-phototronic effects by which carrier transport properties and barrier height could be effectively modulated under the applied mechanical stimulus [31,33,66,67,68,69,70]. Thus, these sensors provide a new avenue for performance enhancement and multifunctional sensing.

Due to the unique piezoelectric and semiconductor properties, ZnO is widely studied in various sensors [18,32,35,36,56,57,61,70,71]. Furthermore, the co-exist of these two properties provide a necessary condition for fabricating piezotronic and piezo-phototronic devices. In 2013, Caofeng Pan demonstrated the first nanowire LED array (Figure 5d,e) [34], which is based on n-ZnO nanowire/p-GaN, for high-resolution pressure mapping with an ultrahigh spatial resolution of 2.7 μm, corresponding to 6350 dpi pixel density. The remarkable sensing performance is derived from the increased recombination rate of electronics and holes at the GaN/ZnO interface by piezo-phototronic effect. Based on similar structure, as shown in Figure 5f, another LED array comprised of PEDOT:PSS and CdS nanorods is fabricated [39]. Owing to the smaller diameter of CdS nanorods, a higher spatial resolution of 1.5 μm is achieved (Figure 5g,h).

Besides LED pressure/strain sensor, piezo-phototronic-effect-enhanced photodetectors is demonstrated. Xun Han et al. fabricated an UV photodetector array based on vertically aligned ZnO nanowires, as shown in Figure 5i [35]. The photodetector array consists of 32 × 40 pixels with the spatial resolution of 100 µm, and each pixel is made of ZnO nanowires and Au electrodes, which are used to form Schottky contact with ZnO nanowires. Owing to the reduced Schottky barrier height by piezo-phototronic effect, carrier transport across the Au/ZnO interface is improved (Figure 5j). As a result, the photoresponsivity and sensitivity are enhanced by 700% and 600% under the applied pressure, respectively.

The same group later reported a piezoelectric pressure sensor [61] that visually recorded and stored pressure distribution by integrating a vertical ZnO nanowires array with a WO_3_ film array that can be colored by the current through it. Because of the pressure-induced piezopotential at the electrode/ZnO interface, the current of each pixel is tuned by piezoelectric effect, leading the coloration of pixels in WO_3_ film array. Therefore, the abilities of pressure detection, recording, and storage are integrated monolithically.

Monitoring the composition of gases in the air is important to safety [28,71,72,73,74]. Ranran Zhou et al. demonstrated a ZnO micro/nanowire-based gas sensor for H_2_ and NO_2_ detection that is enhanced by piezotronic effect and is also sensitive to compressive strain [36]. To fabricate the sensor, a single ZnO micro/nanowire is transferred onto the polystyrene (PS) substrate, with the ZnO c-axis parallel to the longitudinal axis of rectangle substrate. Then, the two ends of the micro/nanowire are fixed by sliver paste that also serves as electrode. According to the I–V curves, with the increased H_2_ concentration, the current increases, while with the increased NO_2_ concentration, the current decreases. At the same time, the I–V curves verify that the single Schottky junction is formed at the M-S contact interface, and the current is significantly increased by compressive strains, which leads to the output detection current that is improved by 238.8% and 5359% for NO_2_ and H_2_, respectively. The performance enhancement is attributed to piezotronic effect. As shown in Figure 5k,l, with the compressive strain along the crystal orientation of ZnO (c-axis), positive piezoelectric polarization charges are induced at the M-S contact interface, and the barrier height is reduced, which results in the larger current.

## 4. Multifunctional Sensors Based on 2-D Materials

The discovery of graphene and its fascinating properties opens up new areas of research on 2-D materials [41,74,75,76,77,78,79]. As many pioneering achievements are reported in the past few decades, tremendous efforts are devoted to 2-D materials-based multifunctional sensors. According to the construction strategy, 2-D materials-based multifunctional sensors can be primarily classified into two categories. One is the sensors fabricated from conductive composite employing 2-D materials as fillers or coating [47,53,80,81], similar to the counterparts based on 0-D and 1-D materials. Another is the sensors based on mono- or few-layer 2D nanosheets [20,40,66,82,83,84]. The former mainly involves piezoresistive conductive structure. While the latter usually involves heterojunction in which piezoelectric polarization charges can be induced at the heterojunction interface by strain or pressure, multifunctional sensing is achieved by piezo-phototronic effect.

2-D materials take different advantages depend on the distinct device structures and transmission mechanisms of the two categories mentioned above. Firstly, when 2-D materials are adopted to build conductive composites, the facile synthesis in solution and easy dispersion in matrixes provide great convenience for device construction; furthermore, the larger plane size and overlapping area than 0-D and 1-D materials serves to create more robust conductivity under tensile strain, and these merits facilitate the exploration of piezoresistive multifunctional sensors based on 2-D materials. Secondly, when 2-D mono- or few-layers are employed in heterojunction multifunctional sensors, the nature of dangling-bond-free surfaces break through the limitation on lattice matching for fabricating heterojunction [20,84]. Moreover, direct bandgap and piezoelectricity, which are exclusive features of mono-layers due to the layer-dependent property, serve to create excellent photodetection and outstanding response to mechanical signals. Furthermore, as for 2-D few-layers, despite the indirect bandgap and non-piezoelectricity, the high mobility of 2-D channel is also attractive for high-performance sensors. These unique advantages prompt researchers to design various sensors that bring superiority of 2-D materials into full play.

### 4.1. 2-D Materials Based Conductive Composites

As representative 2-D materials, graphene possesses outstanding conductivity, mechanical strength, and stability. However, it is still not easy to prepare high-quality graphene in large quantities. Compared with graphene, rGO can be considered as defective graphene, and it can be synthesized by facile and low-cost solution methods with potential of mass production. Beneficial for the synthesis process in solution, rGO can be employed easily in sensors based on conductive composites, similar with 0-D and 1-D conductive fillers, as stated previously. For example, by coating rGO on cellulose acetate (CA) fiber bundles, a conductive composite is prepared based on rGO. Then, the conductive bundles are attached onto a stretchable tape to fabricate a multifunctional mechanical sensor, with the fiber arrangement perpendicular to the strain direction. As a strain sensor, it exhibits a sensitivity of −8.9 and a detection limit of 0.05%. When used as a pressure and vibration sensor, the detection limit is 2 Pa and 8 µm, respectively [80].

Both higher sensitivity and broader stretchable range are strongly desired for mechanical sensors. However, higher sensitivity depends on distinct structure deformation, whereas broader stretchable range requires the conductive pathways are maintained [47]. To achieve outstanding sensitivity and stretchability at the same time, Yina Yang et al. constructed a hybrid network comprised of Ti_3_C_2_T_x_ MXene nanoparticles and nanosheets [47]. The sensor is fabricated by coating the mixture of Ti_3_C_2_T_x_ nanoparticles and nanosheets on the PDMS substrate. With applied strain, Ti_3_C_2_T_x_ nanoparticles move away from each other, while Ti_3_C_2_T_x_ nanosheets keeps the cracks of the conductive film in a small magnitude and bridges the nanoparticles to maintain the conductive pathways, which endow the sensor with a wide sensing range of 0–53%. Because of the migration of nanoparticles, a detection limit of 0.025% is obtained, and the sensor demonstrates the ultrahigh sensitivity of 178.4 within 5% strain, 505.1 for 5–35% strain, and 1176.7 for 35–53% strain.

### 4.2. Multifunctional Sensors Based on 2-D Materials and Heterojunctions

With the non-centrosymmetric structure, 2-D piezoelectric monolayers have become emerging staring materials [37,38,69,70]. Moreover, due to the atomically flat surface, 2-D materials possess the ability of constructing a rich variety of heterojunctions with the help of van der Waals (vdW) force [6,20,66,67,85].

**Figure 6:**
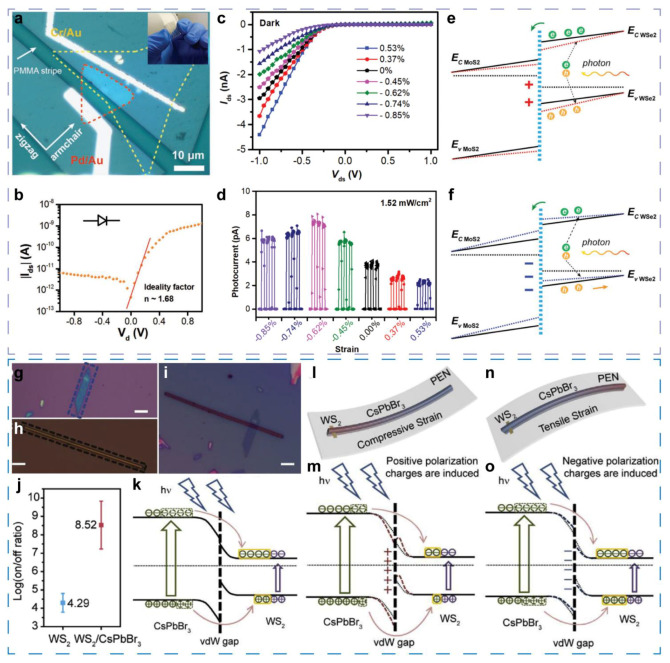
Multifunctional sensors based on 2-D materials. (**a**) Optical image of the MoS_2_/WSe_2_ vdWH photodiode. (**b**) I-V curve of the MoS_2_/WSe_2_ vdWH diode. (**c**) I-V curve of the MoS_2_/WSe_2_ vdWH photodiode in dark with different strains. (**d**) Strain-dependent photocurrent of the MoS_2_/WSe_2_ vdWH photodiode under the illumination at applied bias of 0 V. (**e**,**f**) Schematic illustration of the heterojunction energy band with positive and negative piezopolarization induced by strain, respectively. Reproduced with permission. [67] Copyright 2018, Wiley-VCH. (**g**,**h**) Optical image of the chosen WS_2_ flake and CsPbBr_3_ nanowire for constructing a vdWH, respectively. (**i**) Optical image of the as-assembled WS_2_/CsPbBr_3_ vdWH. (**j**) Statistics the maximum on/off ratio. (**k**) Schematic diagram of the transfer of light-induced carrier from CsPbBr_3_ to WS_2_. (**l**,**m**) Schematic diagram of the vdWH and energy band under compressive strain, respectively. (**n**,**o**) Schematic diagram of the vdWH and energy band under tensile strain, respectively. Reproduced with permission. [82] Copyright 2019, Elsevier.

Pei Lin et al. fabricated a vertically stacked van der Waals heterostructure (vdWH) consisting of an n-MoS_2_ monolayer and a p-WSe_2_ few-layer on PET substrate [67]. Figure 6a depicts the optical image of the device. The PET is used because of the flexibility capable to induce strain in the heterojunction. I–V curve of the p-n junction without light illumination and strain reveals the current rectification behavior, and an ideality factor of 1.68 is obtained, as shown in Figure 6b. With the strain induced, the current is tuned effectively (Figure 6c), due to the piezotronic effect, where the band structure is affected by strain induced-piezopotential. Then, under 532 nm optical illumination with 1.52 mW cm^−2^ power density (Figure 6d), the photo-current is increased by 86% under −0.62% strain compared with the strain-free state, and the maximum photoresponsivity achieves about 3.4 mA W^−1^. The great improvement is attributed to the facilitated separation and transport of light-induced carriers by the titling of local interface band according to piezo-phototronic effect. As shown in Figure 6e,f, strain-induced positive piezopolarization charges render the energy band of WSe_2_ slope steeper, and the induced negative piezopolarization make the WSe_2_ energy band gentle. Therefore, the compress strain, which induces positive piezopolarization charges at the interface, accelerates the separation of photoinduced excitons, and the photocurrent is improved. However, a further increased compress strain leads to a downward slope in energy band of MoS_2_, which will trap electrons and decrease the photocurrent. In general, owing to the piezotronic and piezo-phototronic effect, this MoS_2_/WSe_2_ heterojunction flexible sensor exhibits the ability of sensing strain and the strain-enhanced photodetection performance. The same group also reported a WSe_2_/CdS vdW p-n heterojunction [66]. For this sensor, the photocurrent is increased by 110% with −0.73% strain, and the photoresponsivity up to 33.4 A W^−1^ is achieved.

These above outstanding research achievements demonstrate the great facilitation of piezotronic and piezo-phototronic devices for developing multifunction-integrated sensors again. However, only mono-layer can provide piezopotential due to the centrosymmetric structure of bilayers and bulk crystals [37,38]. It is not easy to obtain 2-D mono-layers by mechanical cleavage or CVD method [86,87], and 2-D monolayers synthesized by solution process usually have more defects and weaker crystal quality [87], which are disadvantageous for vdWH construction [84,85,88]. It seems that 2-D few-layer materials cannot be used to fabricated piezotronic or piezo-phototronic devices. Fortunately, 1-D piezoelectric materials can also induce piezopotential at the interface in vdWH [36,66], and thus the strict restriction that piezotronic and piezo-phototronic devices cannot be fabricated from few-layer 2-D materials is removed.

According to this line of thought, a WS_2_/CsPbBr_3_ vdWH planar photodetector [82] is demonstrated by precisely transferring a CsPbBr_3_ nanowire (Figure 6h) to the target WS_2_ nanoflake (Figure 6h). Figure 6i demonstrates the as-assembled vdWH. This work successfully combined the high mobility of WS_2_ nanoflakes with the outstanding optoelectronic properties of CsPbBr_3_ nanowires. Due to depletion of 2-D WS_2_ channel in the vdWH area, the dark current is effectively suppressed. At the same time, the photocurrent is increased by the effect transfer of light-induced carriers from CsPbBr_3_ nanowires to WS_2_ nanoflakes (Figure 6k). As a result, an ultrahigh on/off ratio up to 10^9.83^ is obtained (Figure 6j). In addition, the highest responsivity of 57.2 A W^−1^ and detectivity of 1.36 × 10^14^ Jones are achieved at a low V_d_ of 2 V, respectively. When strain is applied to the photodetector, the photocurrent can be tuned by a factor of 11.3, revealing the function of detecting strain. Owing to the unprecedented devices architecture in which the channel is perpendicular to the heterojunction stack direction, the modulation of transfer of light-induced carriers across the interface is enabled by piezo-phototronic effect. As shown in Figure 6l,m, when positive polarization charges are generated at the interface by compressive strain, the barrier is reinforced for carrier transfer, leading to the reduced photocurrent. With negative polarization charges generated at the interface (Figure 6n,o), the barrier is weakened, resulting in the improved carrier transfer and photocurrent.

## 5. Conclusions and Outlook

In summary, a comprehensive review is conducted on multifunctional sensors fabricated from low-dimensional materials. After a brief representation about characteristics of low-dimensional materials and the relevant main sensing mechanisms, 0-D, 1-D, and 2-D materials-based multifunctional sensors are divided and introduced systematically, according to their primary sensing functions, material action in the sensors, device architecture, and transmission mechanisms. Detection of multiple mechanical signals, such as strain, pressure, and bending, as well as environmental information, such as temperature, light, and gas component, can be carried out by a multifunctional sensor. In addition, the integrated ability of power supplying and information expression and storage is also demonstrated in some recent reports. The optimization of matrix structure, device architecture, and synergistic effect between different low-dimensional materials can effectively improve the performance of sensors based on active composites, and new process technologies, such as 3D/4D printing, are accelerating the evolution of these sensors. New emerging areas, such as piezotronics and piezo-phototronics, offer better performance and integrated strain/pressure sensing ability to sensors fabricated from piezoelectric semiconductors, greatly expending the research approach for multifunctional sensors. With the prosperous development of low-dimensional multifunctional sensors, richer functions and stronger performance are being implemented.

Despite the remarkable achievements in the development of multifunctional sensors, tremendous efforts are still needed to promote the further development of this field. The first challenge for these advanced multifunctional sensors is that they are difficult to be mass-produced. Some low-dimensional carbon materials, such as CB and CNTs, exhibit great advantages of mass-production after a long time of development [17,89], but many novel low-dimensional materials, such as mechanically exfoliated 2-D mono/few-layers, are still in a stage where they only can be synthesized in small amounts at laboratory level [85,87,90]. Solution methods are widely used for synthesizing nanomaterials, but it has been proven that the as-prepared samples usually possess abundant defects, especially for 2-D materials [78]. CVD is a promising method for controlled growth of high-quality nanocrystals, such as 1-D nanowires and 2-D mono-layers, in large quantities, but the insufficient quality stability hinders the commercial and standardized production. Besides, many assembly processes of these sensors are also different to be employed in mass-production. Some of these advanced sensors are fabricated by emerging methods, such as 3D/4D printing that use homemade special ink and precise manual transfer of nanoscale materials, and these methods can be carried out by experimental sets but are not suitable for production lines before necessary optimization [18,27,58,66,67]. Fabric sensors may possess better opportunity for mass-production due to the compatibility with textile process and the relatively facile fabrication only if the bottleneck of durability is broken [56,59,89]. However, fabric sensors are only a few of the various advanced multifunctional sensors. The second challenge is that many of these sensors are incompatible with semiconductor processes. Up to now, the whole system of smart devices and networks has been mainly based on silicon, and it will be better that the advanced multifunctional sensors serve for and be a part of the silicon world. Unfortunately, most of these sensors are fabricated from non-silicon materials, or the assemble processes cannot cooperate with the current semiconductor processes [10,11,30,56,67]. Thus, the exploration of integrating these sensors with silicon-based chips has been a popular topic. Developing carbon-based technology and the relative interdisciplinary frontier may be an effective approach for constructing a new type of smart system and giving full play to advantages of these sensors in the future, but it has a long way to go. The third challenge, we think, is the integration of functions more than sensing, especially the function of powering. There are few works that can perform all the functions of powering, calculating, and actuating by one device or monolithically integrated system. Self-powered sensors have exhibited the ability of powering, but actuating or other functions are not integrated. One main reason is that the energy consumption of actuating and calculating is much larger than the energy that can be supplied by current self-powering devices. Thus, more powerful energy supplying of self-powered systems is expected. Furthermore, self-powered systems usually convert ambient energy into electrical energy, and ambient energy is unstable because of the random and continuous change of environmental conditions [2,4]. It is necessary to develop an energy management chip that is suitable for emerging energy, such as nanogenerator and other environmental energy. However, mainly due to the instability, the efficiency of energy management for random environment energy is low. Besides improving energy supplying in the self-powered system, reducing energy consumption of calculating and actuating is also a significant approach, in which the interdisciplinary frontier is explored. In general, looking beyond multifunctional sensors and forward to self-powered systems, the fascinating prospect calls for the joint efforts of researchers from different fields.
